# Long non-coding RNA SOX2OT in tamoxifen-resistant breast cancer

**DOI:** 10.1186/s12860-024-00510-y

**Published:** 2024-04-22

**Authors:** Jeeyeon Lee, Eun-Ae Kim, Jieun Kang, Yee Soo Chae, Ho Yong Park, Byeongju Kang, Soo Jung Lee, In Hee Lee, Ji-Young Park, Nora Jee-young Park, Jin Hyang Jung

**Affiliations:** 1 Department of Surgery, School of Medicine, Kyungpook National University, Daegu, Republic of Korea; 2 Cell & Matrix Research Institute, Kyungpook National University, Daegu, Republic of Korea; 3 Department of Oncology/Hematology, School of Medicine, Kyungpook National University, Daegu, Republic of Korea; 4 Department of Pathology, School of Medicine, Kyungpook National University, Daegu, Republic of Korea; 5 Kyungpook National University Chilgok Hospital, Hoguk-ro 807, Buk-gu, 41404 Daegu, Republic of Korea

**Keywords:** Breast cancer, Tamoxifen, Resistance, LncRNA, SOX2OT

## Abstract

Hormone receptor (HR)-positive breast cancer can become aggressive after developing hormone-treatment resistance. This study elucidated the role of long non-coding RNA (lncRNA) SOX2OT in tamoxifen-resistant (TAMR) breast cancer and its potential interplay with the tumor microenvironment (TME). TAMR breast cancer cell lines TAMR-V and TAMR-H were compared with the luminal type A cell line (MCF-7). LncRNA expression was assessed via next-generation sequencing, RNA extraction, lncRNA profiling, and quantitative RT-qPCR. SOX2OT overexpression effects on cell proliferation, migration, and invasion were evaluated using various assays. SOX2OT was consistently downregulated in TAMR cell lines and TAMR breast cancer tissue. Overexpression of SOX2OT in TAMR cells increased cell proliferation and cell invasion. However, SOX2OT overexpression did not significantly alter SOX2 levels, suggesting an independent interaction within TAMR cells. Kaplan–Meier plot analysis revealed an inverse relationship between SOX2OT expression and prognosis in luminal A and B breast cancers. Our findings highlight the potential role of SOX2OT in TAMR breast cancer progression. The downregulation of SOX2OT in TAMR breast cancer indicates its involvement in resistance mechanisms. Further studies should explore the intricate interactions between SOX2OT, SOX2, and TME in breast cancer subtypes.

**Supplementary Information:**

The online version contains supplementary material available at 10.1186/s12860-024-00510-y.

## Introduction

Hormone receptor (HR)-positive breast cancer constitutes a prevalent subtype characterized by the presence of estrogen receptor (ER) and/or progesterone receptor (PR) positive cells, enabling hormonal stimulation of cancer growth [1, 2]. HR-positive breast cancer accounts for approximately 60–70% of cases and treatment primarily relies on hormone therapy, including drugs like tamoxifen and aromatase inhibitors, with targeted therapies such as CDK4/6 inhibitors sometimes used [3, 4]. HR-positive breast cancer is generally associated with a better prognosis than triple-negative breast cancer (TNBC) or HER2-positive breast cancer [5–7]. This subtype can be managed with hormone treatments that either suppress hormones or inhibit HRs [8–10]. However, not all breast cancer cells may respond consistently, and those resistant to hormonal treatment can exhibit more aggressive characteristics, making treatment more challenging [11–14].

Long non-coding RNA (lncRNA) is a class of RNA molecules implicated in cancer development through various mechanisms, which is extensively transcribed in both human and mouse genomes. Characterized as a non-protein coding transcript longer than 200 nucleotides, lncRNA has been reported to play a significant role in cancer development and proliferation by acting as gene expression regulatory factor [15, 16]. They can regulate gene expression by binding to DNA, affecting epigenetic modifications, and interacting with chromatin-modifying proteins, thereby altering the expression of genes involved in cell proliferation, apoptosis, and DNA repair [17, 18]. LncRNAs can also engage in RNA-protein interactions, acting as miRNA sponges or influencing post-transcriptional and post-translational regulation, resulting in the dysregulation of key oncogenes or tumor suppressor genes. Additionally, some lncRNAs can contribute to chromosomal instability, influence alternative splicing, and modulate signaling pathways involved in cancer progression. However, it is crucial to note that not all lncRNAs have pro-oncogenic roles, as some may act as tumor suppressors. The specific mechanisms and functions of lncRNAs in cancer development can vary among different cancer types and subtypes, making them a subject of active research with potential therapeutic implications.

Tamoxifen has been the gold standard treatment for HR-positive breast cancer for decades [19, 20], and researchers have been attempting to identify genes or genomes specifically expressed in tamoxifen-resistant breast cancer and incorporate them into treatment mechanisms. Some lncRNAs, including HOX transcript antisense RNA (HOTAIR) and Thymopoietin antisense transcript 1 (TMPO-AS1), have been shown to contribute to endocrine therapy resistance in breast cancer through distinct mechanisms [21–24]. One of the lncRNA, SOX2 has been identified in 43% of basal cell-like breast cancers [25], and it is significantly associated with CK5/6, EGFR, and vimentin immunoreactivity, while showing an inverse association with estrogen and progesterone receptor status [26]. Although SOX2 mechanism remains largely elusive, several studies have indicated that SOX2 and SOX2OT are co-expressed at similar locations. Furthermore, SOX2OT appears to play a role in SOX2 regulation. However, recent studies have reported that lncRNAs do not simply affect the tumor cells; instead, cancer promotion, proliferation, and drug resistance is involved through more complex interactions with the tumor microenvironment (TME) [27–29].

This study aimed to verify the biologic role of SOX2OT associated with SOX2 in tamoxifen-resistant breast cancer and to understand the potential relationship between TME and lnRNA SOX2OT.

## Materials and methods

### Cell culture

Luminal type A cell line (MCF-7) was purchased from the American Type Culture Collection (ATCC, Manassas, VA, USA). Two different types of tamoxifen-resistant breast cancer cell lines were prepared. One cell line, MCF-7 Tam1 (CRL-3435TM; TAMR-H), was purchased from ATCC, and the other, a tamoxifen-resistant cell line (TAMR-V), was provided and authenticated by the University of Virginia Hospital [30, 31]. The TAMR-V has been continuously exposed to tamoxifen for more than 10 years to maintain its resistance. MCF-7 cells were grown in Dulbecco’s Modified Eagle’s Medium (DMEM; Gibco, Grand Island, NY, USA) supplemented with 10% fetal bovine serum (FBS; Gibco), and TAMR-V cells were maintained in DMEM containing 10% FBS and 10^− 7^ mol/L TAM (Sigma-Aldrich, St. Louis, MO, USA). TAMR-H cells were maintained in DMEM containing 10% FBS, 10-µg/mL human insulin (Sigma), and 1-µM 4-hydroxytamoxifen (Sigma).

Next-generation sequencing (NGS) was performed to distinguish two different types of TAMR cell lines, and the results are presented as a heatmap (Supplementary Fig. 1). All cells were cultured for three days to verify their growth without any treatment, and it was confirmed that they proliferated well (Supplementary Fig. 2).

Profiling and confirmation of lncRNA expression via quantitative reverse transcription polymerase chain reaction (RT-qPCR).

Total RNA was isolated from breast cancer cells using RNAiso Plus (Takara, Otsu, Japan), and its concentration was measured using NanoDrop ND-2000 (Thermo Scientific, Wilmington, DE, USA). For lncRNA expression profiling, 2-µg total RNA was reverse transcribed to cDNA using Human LncProfilers™ qPCR Array Kits (System Biosciences, Mountain View, CA, USA).

To confirm the LncProfilers™ qPCR array results, the total RNA in breast cancer cells was extracted and random hexamer (ELPIS-Biotech Inc., Daejeon, South Korea) was applied, following the manufacturer’s protocols. The expression levels of specific lncRNAs, including SOX2 and SOX2OT, were further confirmed by RT-qPCR. This was performed using both TaqMan Gene Expression Master Mix and Power SYBR Green PCR Master Mix (Applied Biosystems, Foster City, CA, USA).

The relative gene expression was normalized to the housekeeping genes GAPDH and β-actin, employing the 2^−ΔΔCT^ method. Primers and TaqMan probes used were for SOX2 (5′-AAC CCC AGA TGC ACA ACT C-3′, 5′-GCT TAG CCT CGT CGA TGA AC-3′, Hs04234836-s1), SOX2OT (5′-GCT CGT GGC TTA GGA GAT TG-3′, 5′-CTG GCA AAG CAT GAG GAA CT-3′, Hs00415716_m1), GAPDH (5′-ACG GGA AGC TTG TCA AT-3′, 5′-TGG ACT CCA CGA CGT ACT CA-3′, Hs99999903_m1), and for β-actin (5’-TTG CCG ACA GGA TGC AGA A-3’ and 5’-GCC GAT CCA CAC GGA GTA CT-3’). Amplification efficiencies for both TaqMan and SYBR Green methods were tested, and all samples were evaluated in triplicate to ensure accuracy and reproducibility.

### Transformation and transfection

SOX2OT sequences were synthesized by Invitrogen (Thermo Fisher Scientific, Waltham, MA, USA), and SOX2OT cDNA fragments were cloned into the pcDNA 3.1 vector to overexpress SOX2OT (Thermo Fisher Scientific). The empty pcDNA3.1 vector was used as the control and pcDNA-SOX2OT or pcDNA-vector transfection into cells was performed using Lipofectamine™ RNAiMAX (Thermo Fisher Scientific), according to the manufacturer’s protocol.

### 3-(4,5-dimethylthiazol-2yl)-2,5-diphenyltetrazolium bromide assay

Cells were inoculated in a six-well plate (4 × 10^5^/well) for the wound healing assay and in a 96-well plate (5 × 10^3^/well) for the MTT assay. Following culture overnight, cells were transfected using Lipofectamine™ RNAiMAX reagent (Invitrogen) with the transformed specific SOX2OT pcDNA3.1(+) plasmid or pcDNA3.1(+) control plasmid. At 48 h following transfection, the MTT substrate was prepared in a physiologically balanced solution, added to cells in culture typically at a final concentration of 50 µL, and incubated at 37 °C for 4 h. Following removal of the medium, 150-µL dimethyl sulfoxide was added, and the mixture was shaken for 10 min. The quantity of formazan (presumably directly proportional to the number of viable cells) was measured by recording changes in absorbance at 570 nm using microplate spectrophotometer (Agilent Technologies, Winooski, VT, USA). All of the experiments were conducted in triplicate.

### Wound healing assay

To determine the effect on cell migration, TAMR-V and TAMR-H cells were seeded in six-well culture plates and transfected with control pcDNA 3.1(+) or SOX2OT-pcDNA 3.1(+). Subsequently, a line was scratched into the cell monolayer using a sterile pipette tip, and the cells were further incubated. Images were captured at 0-, 48-, and 72-h timepoints using a microscope and camera system (Olympus Co., Tokyo, Japan). Wound closure was measured using the ImageJ wound healing tool (ImageJ. Available online: https://imagej.net/Welcome). The data were representative of three independent experiments.

### Cell invasion assay

For the cell invasion assay, transwell chambers with 8-µm pores were coated with Matrigel (Corning Inc., Tewksbury, MA, USA) and incubated at 37 °C for 4 h, allowing it to solidify. Following transfection, cells were resuspended in DMEM containing 1% FBS and plated in the upper chamber at a density of 1 × 10^4^ cells. The lower chamber contained complete medium supplemented with 10% FBS. Following 48-h incubation, the cells on the internal surface of the chamber bottom were wiped with a cotton swab, fixed with 2% paraformaldehyde, stained with 0.5% crystal violet, and rinsed with phosphate-buffered saline. Four random fields were selected for each culture well under a light microscope, and the number of cells in each view was counted.

### Statistical analysis

Data were presented as means (± standard deviation, SD) of three or more independent experiments. The differences in experimental results between the two groups were analyzed using Student’s t-test, and a statistically significant difference was considered at *p* < 0.05 or *p* < 0.001.

## Results

### SOX2OT was significantly downregulated in both TAMR breast cancer cell lines

**Fig. 1:**
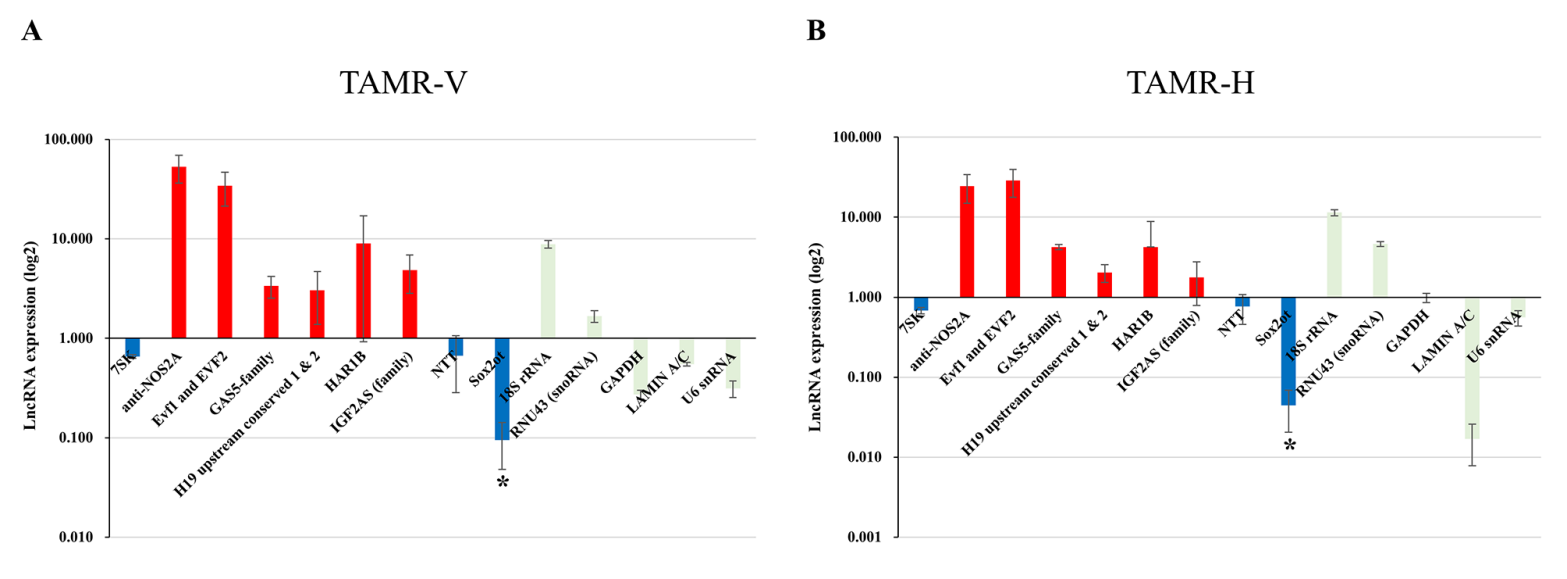
Expression profile of various lncRNAs in tamoxifen-resistant breast cancer cell lines (**A**: TAMR-V; **B**: TAMR-H) compared to MCF7 cells. While 7SK, NTT, and SOX2OT were downregulated in both TAMR breast cancer cell lines, anti-NOS2A, EVF1 and EVF2, GAS5-family, H19 upstream conserved 1 & 2, HAR1B, and IGF2AS family were all upregulated in the TAMR cell lines

Using qPCR array analysis, several lncRNAs were evaluated in TAMR-V and TAMR-H breast cancer cell lines. Among 90 lncRNAs, we extracted transcripts that showed upregulated (> 2-fold) or downregulated (< 0.5-fold) expression, and 24 lncRNAs were analyzed. While lncRNA anti-NOS2A, EVF1 and EVF2, GAS5-family, H19 upstream conserved 1 & 2, HAR1B, and IFG2AS were upregulated, 7SK, NTT, and SOX2OT were commonly downregulated according to the results of each three consecutive tests performed in TAMR-H and TAMR-V compared with MCF-7 (Fig. 1; Table 1). Among nine lncRNA candidates, which were consistently upregulated or downregulated in both TAMR cell lines, SOX2OT was selected on the basis of literature reviews, which showed a strong negative association with TAMR cells [26, 32] (Supplementary Fig. 3).

**Table 1:** Expression profile of the long non-coding RNAs(lncRNAs) expression levels in tamoxifen-resistant breast cancer cell lines (TAMR-V and TAMR-H)

Level of expression	LncRNA^*^	Expression in TAMR-V/MCF7		Expression in TAMR-H/MCF7	
		Average	STDEV	Average	STDEV
Upregulation	anti-NOS2A^*^	52.82983	16.65489	24.41382	9.56836
	EVF1 and EVF2^*^	34.11834	12.70678	28.66012	10.95019
	GAS5-family^*^	3.377255	0.835037	4.2247	0.311261
	H19 upstream conserved 1 & 2^*^	3.027784	1.649675	2.038056	0.510124
	HAR1B^*^	9.013918	8.091384	4.250339	4.595892
	IGF2AS (family)^*^	4.865702	2.040343	1.772124	0.980745
Downregulation	7SK^*^	0.656233	0.028282	0.682445	0.061467
	NTT^*^	0.66975	0.3869	0.767579	0.308688
	SOX2OT^*^	0.094758	0.046917	0.044528	0.023905
Control	18 S rRNA	8.822375	0.791897	11.43136	1.015886
	RNU43 (snoRNA)	1.664141	0.222279	4.614409	0.336516
	GAPDH	0.271698	0.027552	0.983259	0.12848
	LAMIN A/C	0.547854	0.02251	0.016867	0.009027
	U6 snRNA	0.31399	0.060132	0.558524	0.121545

^*^These lncRNAs indicated the reference control

### Expression of SOX2 protein and lncRNA SOX2OT in breast cancer samples from the Kaplan–Meier (KM) plotter

**Fig. 2:**
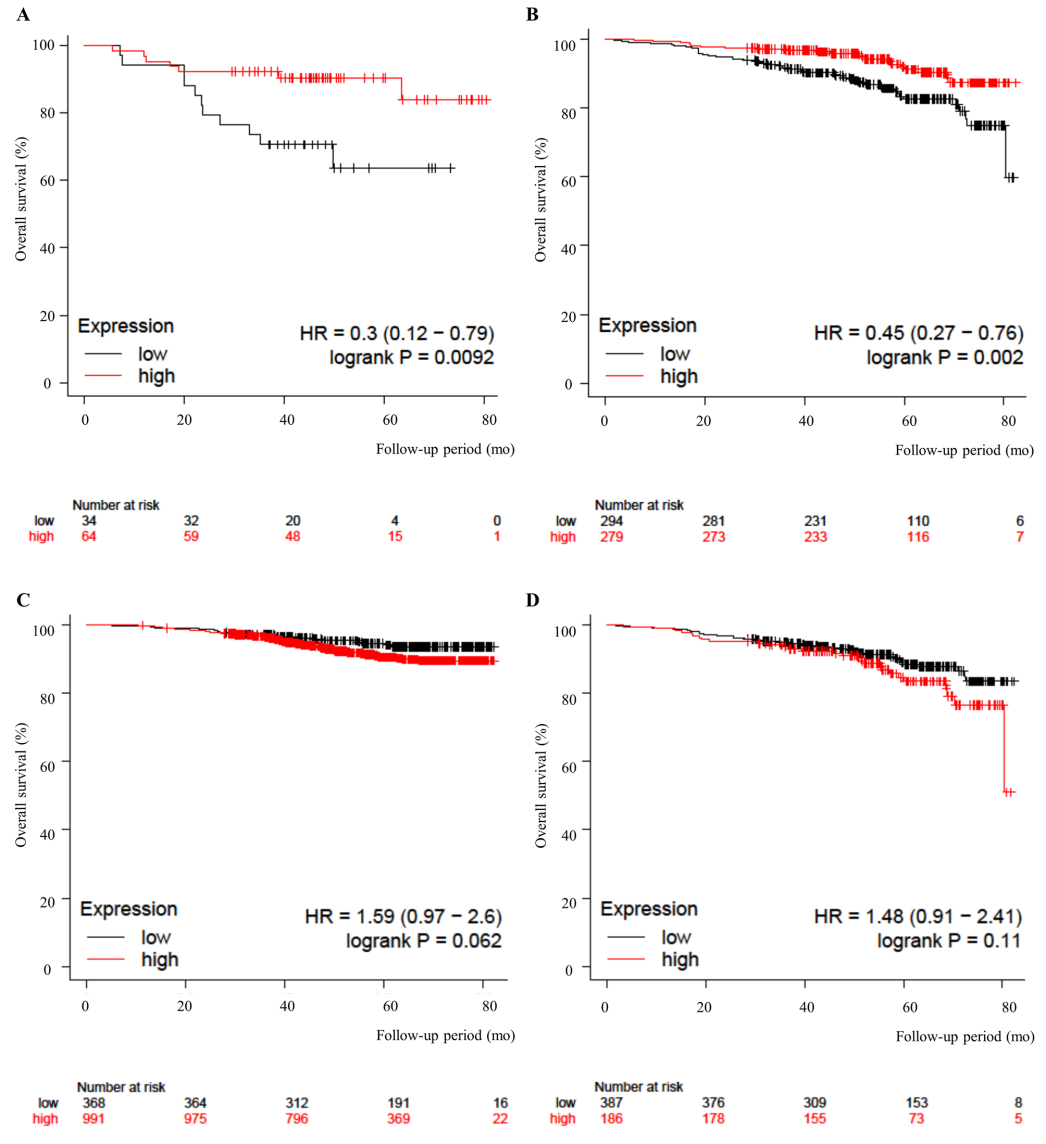
Kaplan–Meier survival curve based on the expression levels of SOX2 and SOX2OT. (**A, B**) Overall survival based on the expression levels of SOX2OT in luminal A and B breast cancer. (**C, D**) Overall survival based on the expression levels of SOX2 in luminal A and B breast cancer

The genome-wide RNA transcript profile from the KM plotter (http://kmplot.com) by RNA sequencing (RNA-seq) dataset in patients with breast cancer was analyzed and noted direct opposite results with SOX2OT and SOX2 in luminal A and B breast cancers. Although lncRNA SOX2OT showed a significantly better prognosis with higher SOX2OT expression in luminal A and B breast cancer (luminal A, HR = 0.30, *p* = 0.0092; luminal B, HR = 0.45, *p* = 0.0020) (Fig. 2A, B), SOX2 showed conversed results without statistical significance (luminal A, HR = 1.59, *p* = 0.0620; luminal B, HR = 1.48, *p* = 0.1100) (Fig. 2C, D).

### TAMR-V and TAMR-H cells were transfected with plasmids pcDNA3.1 and pcDNA3.1 + SOX2OT

**Fig. 3:**
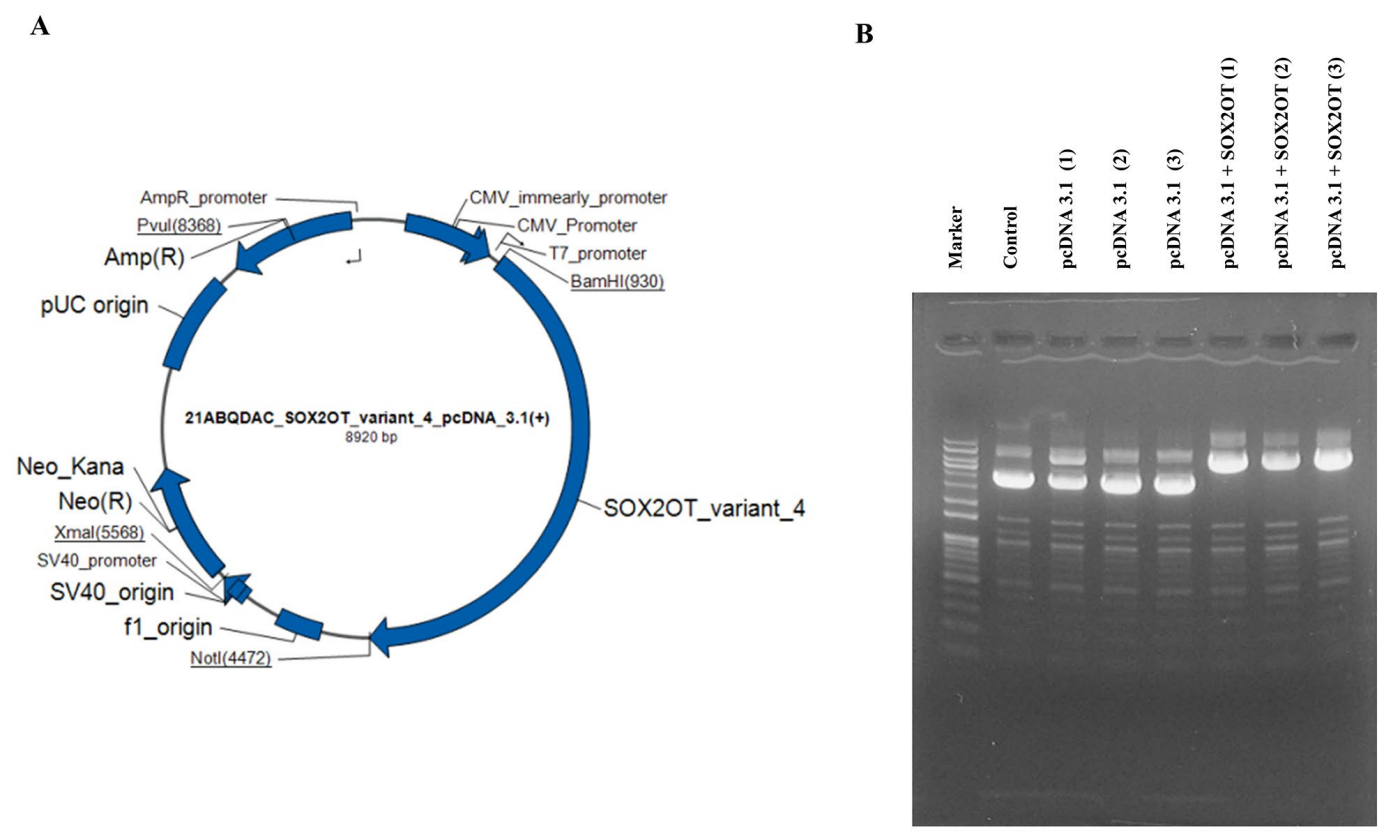
Transfection of pcDNA 3.1 and pcDNA 3.1 with tamoxifen-resistant breast cancer cells. (**A**) Schematic of the SOX2OT_variant_4_pcDNA 3.1(+) vector. (**B**) Agarose gel electrophoresis showed SOX2OT variant inserted pcDNA 3.1(+) plasmid

Since the SOX2OT level was decreased in TAMR cell lines compared with that in the MCF7 cell in previous study, SOX2OT expression was intentionally increased to examine how this reduction affects actual TAMR cancer cells. The SOX2OT expression was transiently increased using SOX2OT transcript introduced pcDNA3.1 plasmid (Fig. 3A), and its transformation was confirmed by agarose gel electrophoresis (Fig. 3B). Both TAMR cells were transfected with pcDNA3.1 control and pcDNA3.1-SOX2OT plasmids, respectively.

**Fig. 4:**
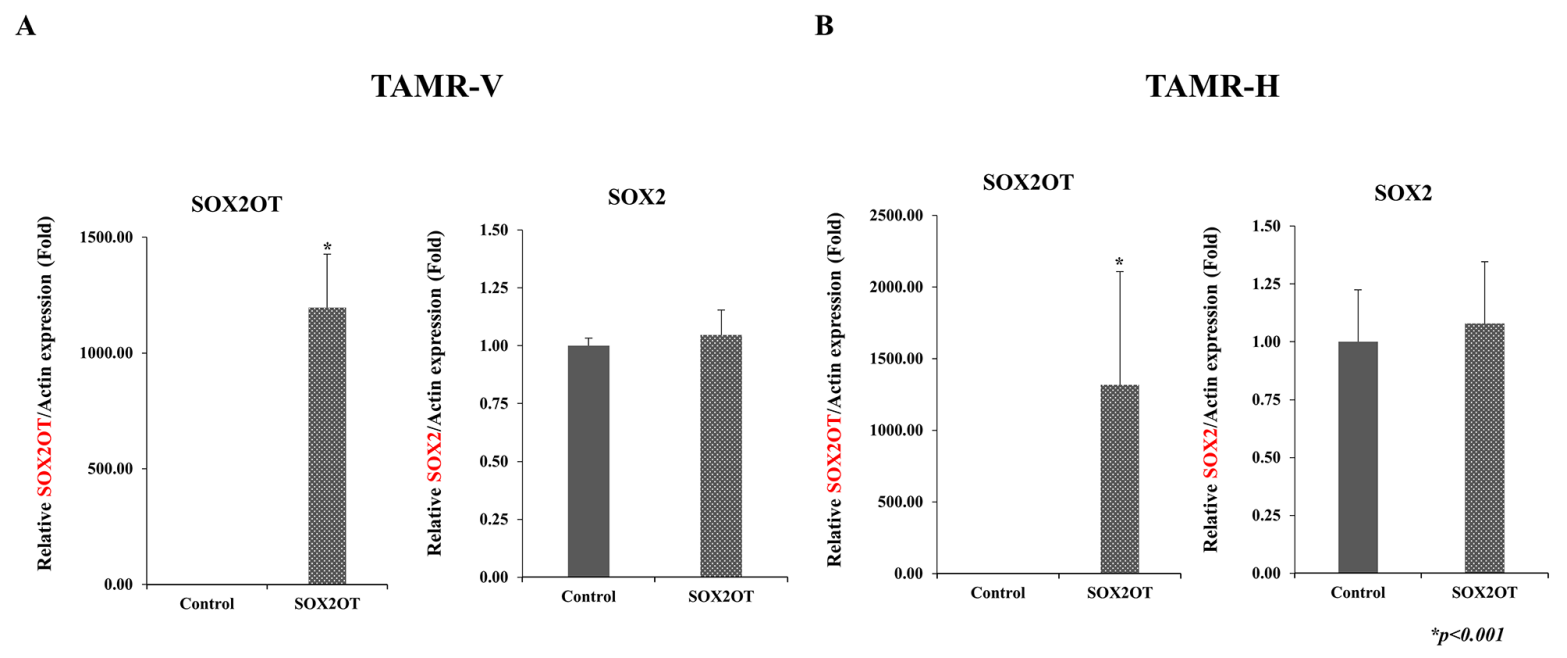
Expression of SOX2OT and SOX2 in TAMR-V (**A**) and TAMR-H (**B**) cells post-transfection. Although SOX2OT expression increased in pcDNA3.1-SOX2OT vector group, SOX2 expression was not affected. Comparison of the two groups was performed using Student’s *t*-test, with error bars representing the standard deviation of at least three independent experiments

The SOX2OT expression level was increased in the pcDNA 3.1-SOX2OT vector group; however, the SOX2 expression level was unaffected (Fig. 4). These results indicate that SOX2OT is not directly involved in SOX2 expression in cell lines, and they interacted independently in TAMR cancer cells.

### SOX2OT overexpression affects cell proliferation in TAMR cells

**Fig. 5:**
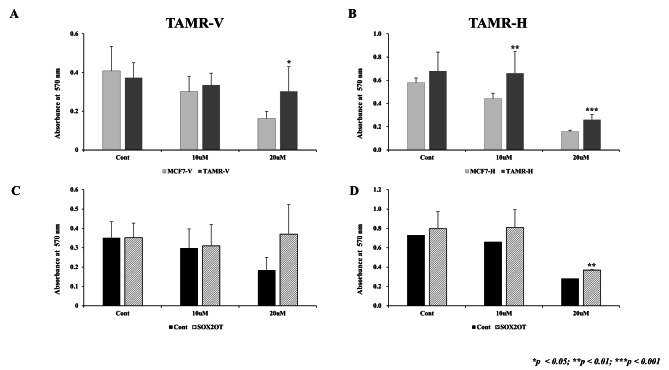
Overexpression of SOX2OT affected cell proliferation in tamoxifen-resistant (TAMR) breast cancer cell lines. (**A, B**) Dose-dependent characterization of tamoxifen resistance in TAMR-V and TAMR-H breast cancer cells. Parental MCF-7, TAMR breast cancer cells were treated indicated concentrations of tamoxifen or tamoxifen-4OH for 48 h. Viable cells were quantified using MTT assay. (**C, D**) TAMR cells were overexpressed using SOX2OT plasmid and then treated with tamoxifen or tamoxifen-4OH. The expression of SOX2OT was observed to be lower in TAMR cells than in control cells, and the cell viability increased when overexpressed again, especially in TAMR-H cells (*p* < 0.001). Comparison of the two groups was performed using Student’s *t*-test, with error bars representing the standard deviation of at least three independent experiments

To assess any differences in SOX2OT expression, both TAMR cell lines were treated with varying doses of tamoxifen and tamoxifen-4OH (control dose [DMSO], 10 µM, and 20 µM). To evaluate the sensitivity and changes in the cell viability of TAMR cells according to the tamoxifen dose, MTT assay was performed. When tamoxifen was treated to MCF-7, TAMR-V, and TAMR-H breast cancer cells to confirm tamoxifen resistance, the MCF-7 cell viability was significantly decreased when 20-µM tamoxifen was treated. However, TAMR-V and TAMR-H cells were not decreased. When SOX2OT was overexpressed, the cell viability of TAMR cells were increased, particularly in TAMR-H cells (*p* < 0.001) (Fig. 5).

### SOX2OT overexpression tends to promote cell invasion but not migration in TAMR cells

**Fig. 6:**
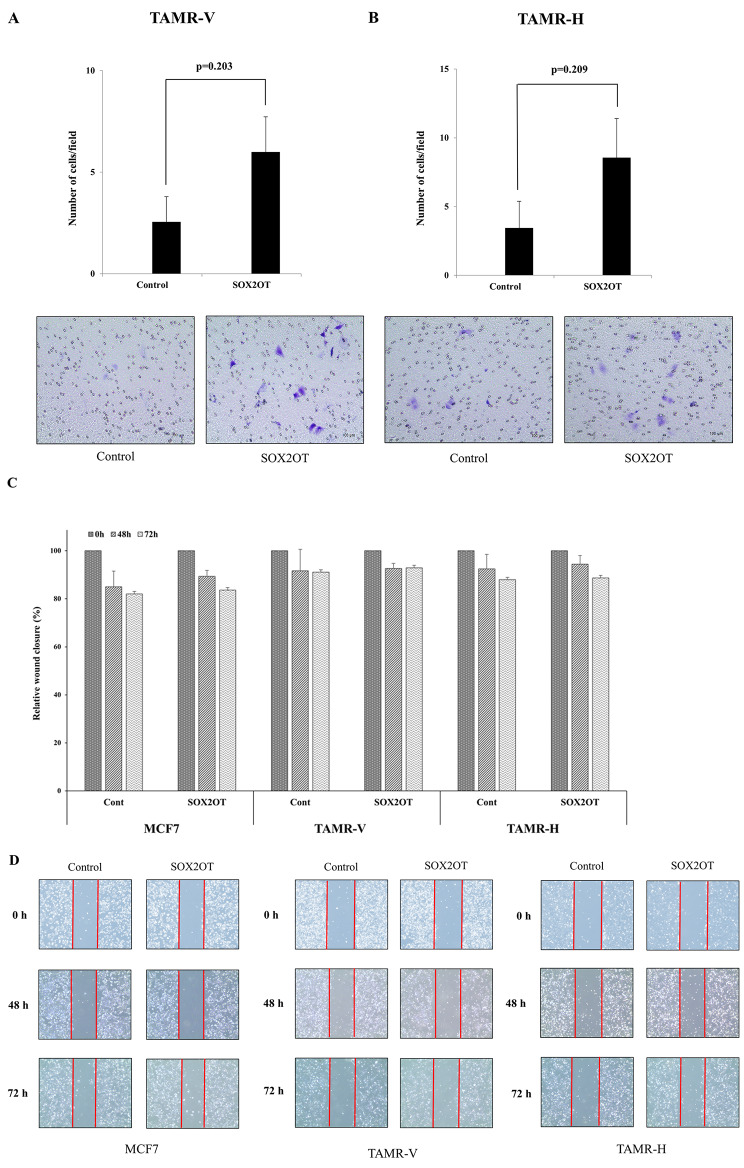
Cell invasion and migration study by SOX2OT overexpression in TAMR breast cancer cell lines. (**A, B**) Cell invasion by Matrigel-coated transwell assay showing a tendency of promotion in TAMR cells, which are transfected with SOX2OT overexpression plasmid or control plasmid. Comparison of the two groups was performed using Student’s *t*-test, with error bars representing the standard deviation of at least three independent experiments. (**C, D**) Migration of MCF7, TAMR-V and TAMR-H cells transfected with SOX2OT overexpression plasmid or control plasmid are detected by wound healing assay

Overexpression of SOX2OT appeared to enhance TAMR cell invasion in the Matrigel-coated transwell assay. Although the difference was not statistically significant, the cells exhibited increased proximity and invasion following SOX2OT overexpression in TAMR-V and TAMR-H cell lines (Fig. 6A, B). Cell movement was tracked for 72 h. No movement was detected in the first 24 h, but a shift in cell movement began at 48 h, becoming more pronounced at 72 h. However, in the wound healing assay used to assess cell migration, TAMR cells demonstrated activity levels almost identical to those of MCF-7 cells (Fig. 6C, D).

### Tissue-specific expression of lncRNA SOX2OT in various breast cancer subtypes

**Fig. 7:**
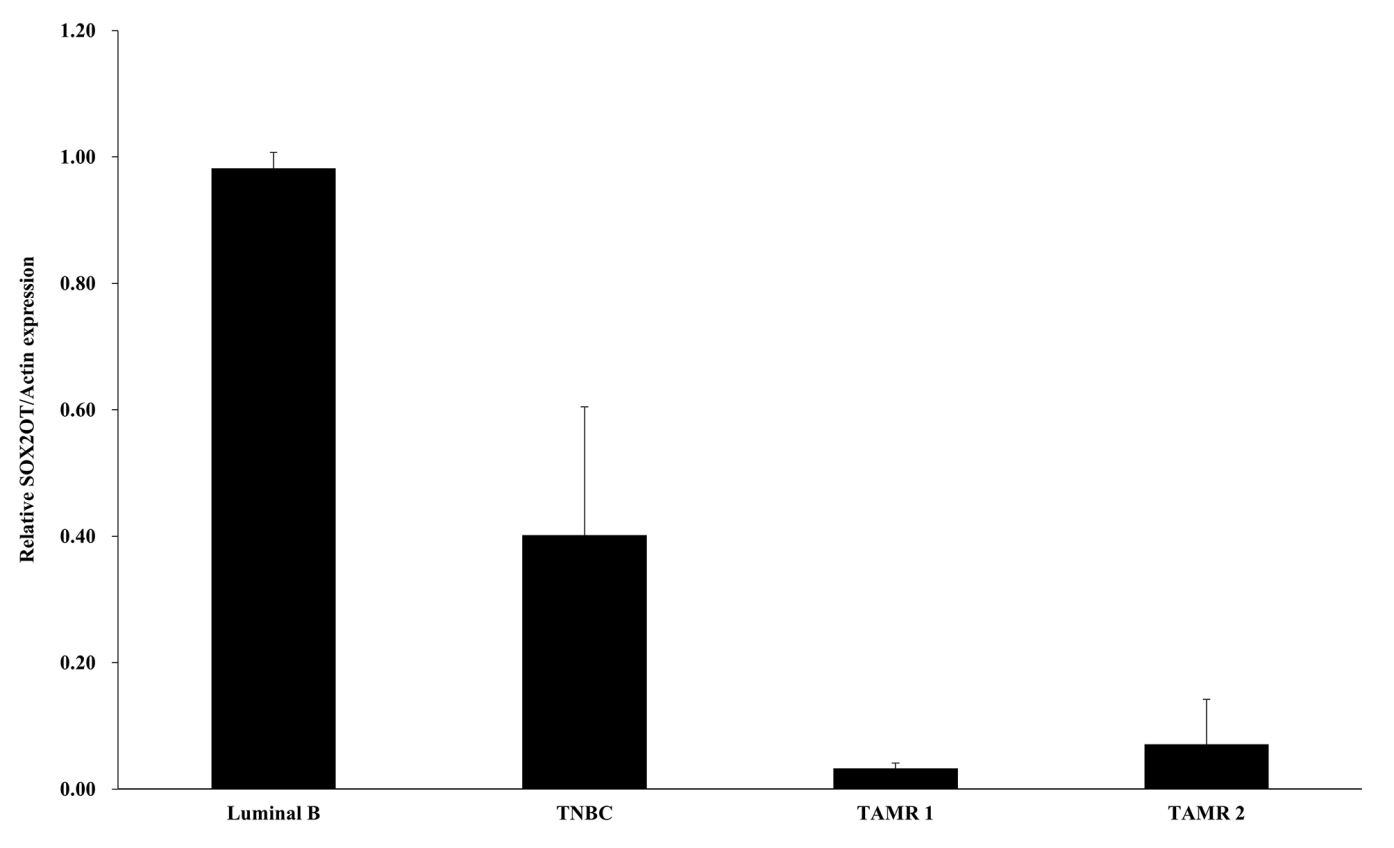
Expression of SOX2OT in patients with breast cancer according to the various subtypes of breast cancer. The expression of SOX2OT was highest in luminal type of breast cancer, and it was lower in TAMR breast cancer than in luminal B type and triple-negative breast cancer

To evaluate the interaction between lncRNAs and tumor cells through the TME, SOX2OT expression was compared in tissues of different breast cancer subtypes. While SOX2OT was highly expressed in luminal B type breast cancer, those in TAMR breast cancers were extremely suppressed (Fig. 7). Therefore, SOX2OT expression was confirmed to be consistently downregulated in both TAMR cell lines as well as TAMR breast cancer tissues.

## Discussion

LncRNAs hold potential as emerging biomarkers and therapeutic targets for breast cancer [33–35]. Notably, several lncRNAs have been associated with the acquired resistance mechanism in HR-positive breast cancer [36, 37]. Understanding their biological roles could shed light on mechanisms to overcome resistance, paving the way for advancements in therapeutic drug development. Moreover, SOX2OT has been identified as a novel biomarker linked to tamoxifen-resistant breast cancer, and its association with SOX2 has also been documented [38–40].

In this study, two different types of TAMR breast cancer cell lines were used. One of TAMR cell lines has been developed by treating with real tamoxifen (TAMR-V) over 10 years, and the other cell line was commercially obtained (TAMR-H) by treating with tamoxifen-4OH. This study utilized multiple cell lines, which, based on the NGS test, exhibited distinct characteristics. Despite the variations in TAMR breast cancer cell lines, SOX2OT consistently showed reduced expression compared to MCF-7. This indicates a potential relationship between SOX2OT and the acquisition of TAMR in breast cancer.

The most significant challenge in the experiment was the sluggish proliferation of HR-positive breast cancer cells [41]. Actual cell proliferation and migration paces were very slow compared with those of TNBC or HER2-positive breast cancer, making it difficult to determine the statistical significance in the results. Therefore, developing a novel drug to overcome hormone-resistant breast cancers is difficult, and HR-positive breast cancers are considered a refractory disease when they achieve hormone resistance [12, 13, 42].

The TAMR-H cell lines displayed a significant increase in tamoxifen-resistant breast cancer proliferation, while TAMR-V cell lines showed a pronounced trend. Furthermore, the addition of SOX2OT worsened cell invasion in both cell lines. In the cell migration test, there was more migration with SOX2OT even if it did not achieve statistical significance. These findings indicate that lower SOX2OT levels in hormone-resistant breast cancer are associated with a better prognosis, similar to HER2-positive or TNBC. Conversely, higher SOX2OT levels correlate with a better prognosis in HR-positive breast cancer, as seen in the KM plot. Our study indicates that while SOX2OT overexpression tends to enhance cell invasion in TAMR breast cancer cells, as evidenced by the Matrigel-coated transwell assay, it does not significantly impact cell migration, as shown in the wound healing assay. This suggests a complex interaction in which SOX2OT selectively influences the invasive capabilities of cancer cells without markedly affecting their migratory behavior. This highlights the nuanced role of lncRNAs in cancer cell dynamics and underscores the need for further investigation into the specific mechanisms by which SOX2OT modulates these distinct aspects of cancer progression.

The addition of SOX2OT did not visibly alter SOX2 levels although SOX2OT levels increased significantly after adding SOX2OT. This suggests a more complex and multifaceted interaction between SOX2OT and SOX2 in tamoxifen-resistant breast cancer. Previous studies suggest that the diverse interactions between SOX2 and SOX2OT plays a crucial role in the progression of breast cancer [32, 43]. Although the proliferation of cells slowed down, making it challenging to observe statistical significance, our cellular experiments demonstrated a clear association between SOX2OT and the proliferation, invasion, and migration of tamoxifen-resistant breast cancer cell lines. Given that these results are consistent not only in cell lines but also in tissue, it can be inferred that SOX2OT can promote tumor growth through interactions with the TME rather than directly affecting the cell lines themselves.

This study provides potential insights into the role of SOX2OT in tamoxifen-resistant breast cancer. We found that SOX2OT expression decreases with tamoxifen resistance, and high levels of SOX2OT enhance the proliferation, invasion, and migration of TAMR cancer cells, yielding poor outcomes. However, achieving statistical significance was impeded by the slow progression of the disease. This made it difficult to conclusively determine the effect of SOX2OT on tamoxifen resistance based solely on our findings, which was a limitation of this study. A further limitation was the differing results of the two TAMR cell types. This suggests an unrevealed mechanism at play rather than a simple difference in tamoxifen type.

## Conclusion

In this study, we confirmed that SOX2OT is downregulated in tamoxifen-resistant breast cancer. Moreover, a higher SOX2OT level is associated with worse outcomes, including increased TAMR cancer cell proliferation, invasion, and migration. However, the slow progression of the disease made it challenging to observe statistical significance. Determining how SOX2OT affects tamoxifen-resistant breast cancer based solely on this study is difficult. To better understand the impact of lncRNA SOX2OT on tamoxifen-resistant breast cancer and the mechanisms behind this resistance, the relationship between SOX2OT and SOX2 should be further investigated in various breast cancer subtypes.

### Electronic supplementary material

Below is the link to the electronic supplementary material.

Supplementary Figure 1: Heatmap of gene expression in TAMR cell lines. Based on the analysis of 144 genes in the two TAMR cell lines that differed more than tenfold from MCF7. Red, black, and green represent higher than average, close to average, and lower than average expressions of a particular gene, respectively

Supplementary Figure 2: Proliferation of breast cancer cell lines without treatment. 1.5 × 10^6^ cells from MCF7, TAMR-V, and TAMR-H breast cancer cell lines were seeded into six wells without treatment, and proliferation was observed over 72 hours, during which the cells all proliferated well

Supplementary Figure 3: Relative expression levels of SOX2OT and SOX2 in various breast cancer cell lines

## Data Availability

The datasets generated and/or analyzed during the current study are available in the Zenodo repository, 10.5281/zenodo.10869043.
