# Supplementary figures and images for: Long non-coding RNA SOX2OT in tamoxifen-resistant breast cancer
Source: BMC Mol Cell Biol. 2024 Apr 22;25:12. doi: 10.1186/s12860-024-00510-y (PMC11036730; doi:10.1186/s12860-024-00510-y)

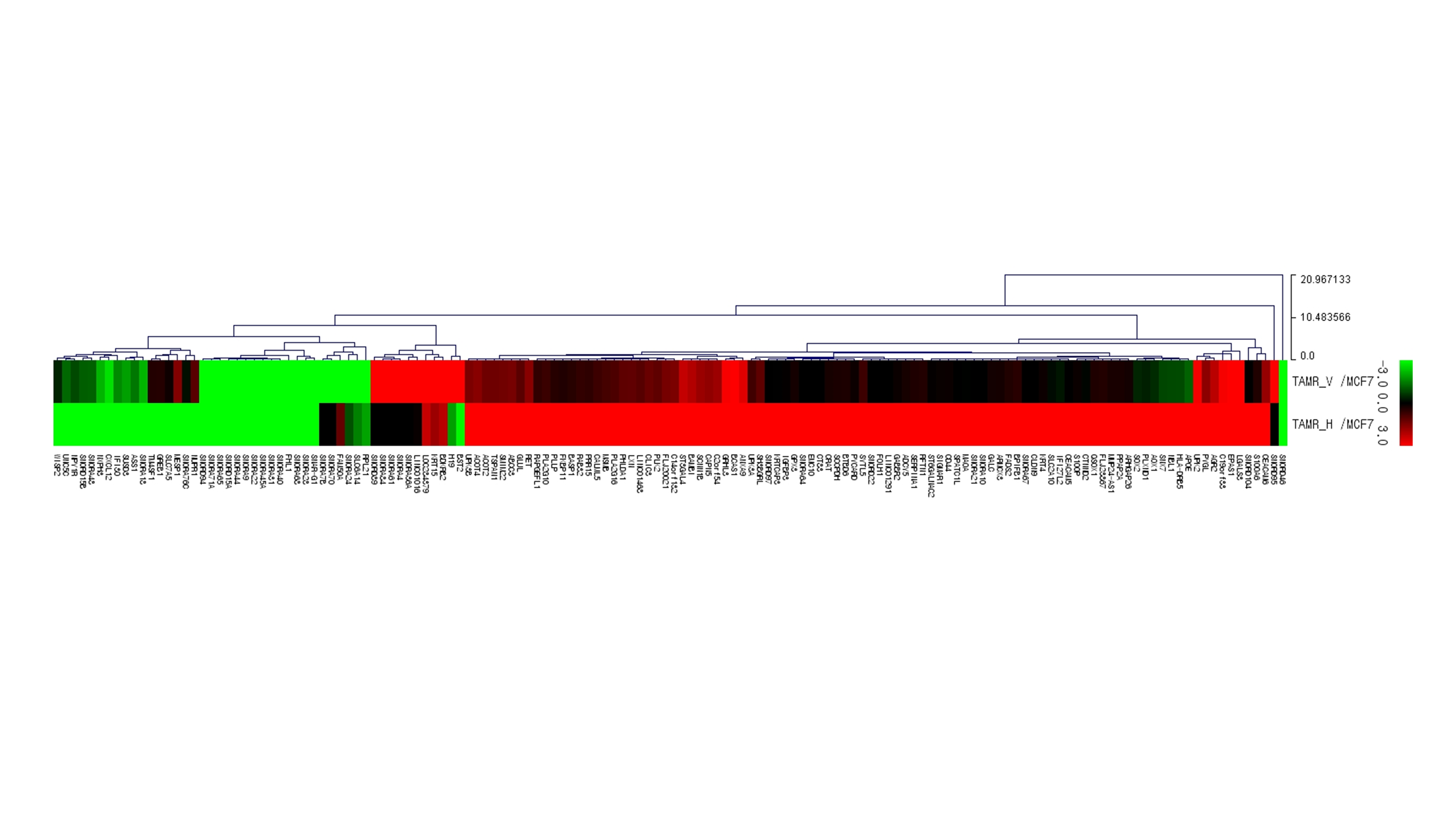

Supplement: Supplementary file 1 — Supplementary Figure 1: Heatmap of gene expression in TAMR cell lines. Based on the analysis of 144 genes in the two TAMR cell lines that differed more than tenfold from MCF7. Red, black, and green represent higher than average, close to average, and lower than average expressions of a particular gene, respectively [file 12860_2024_510_MOESM1_ESM.png]

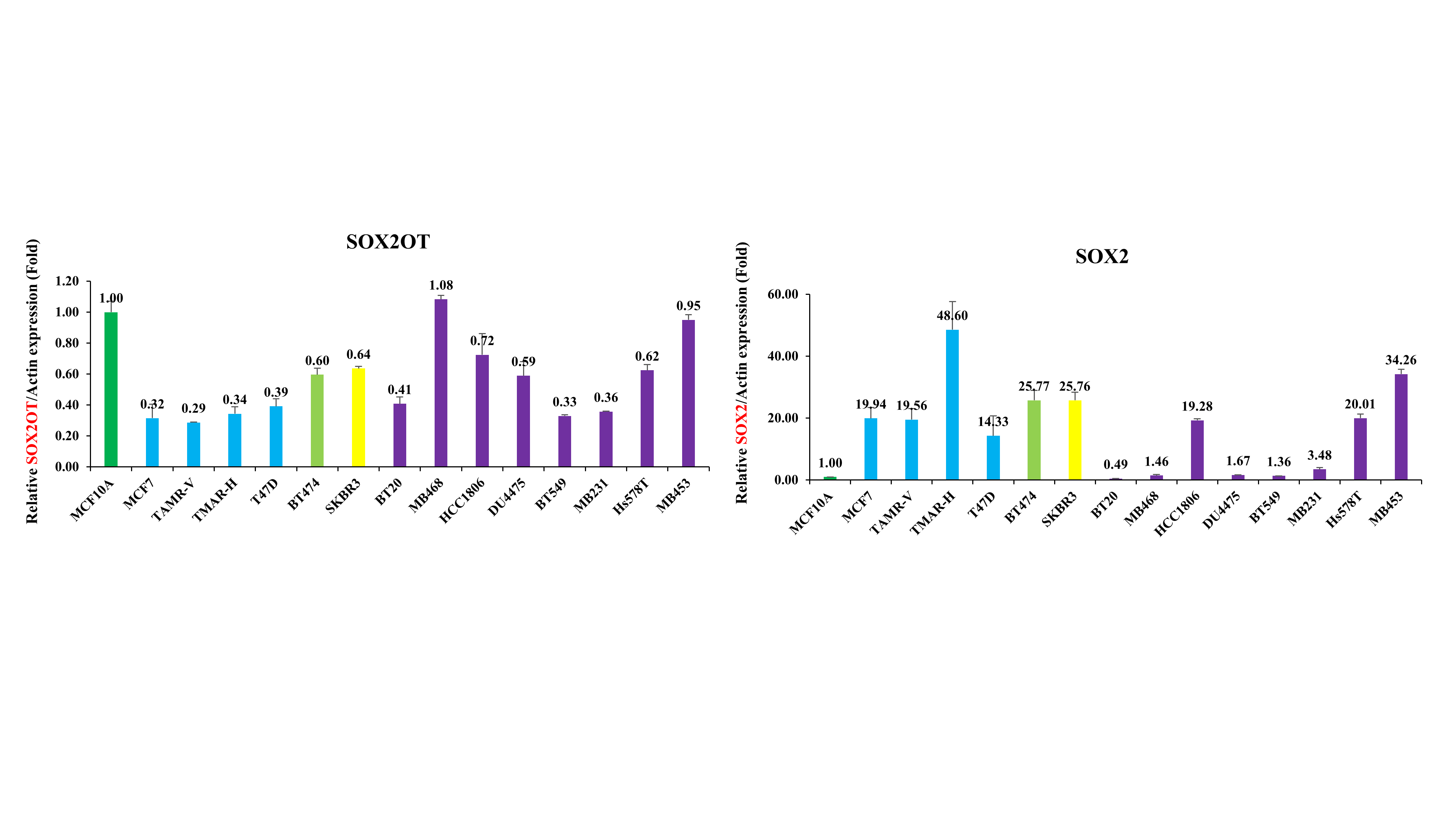

Supplement: Supplementary file 2 — Supplementary Figure 2: Proliferation of breast cancer cell lines without treatment. 1.5 × 10^6^ cells from MCF7, TAMR-V, and TAMR-H breast cancer cell lines were seeded into six wells without treatment, and proliferation was observed over 72 hours, during which the cells all proliferated well [file 12860_2024_510_MOESM2_ESM.png]

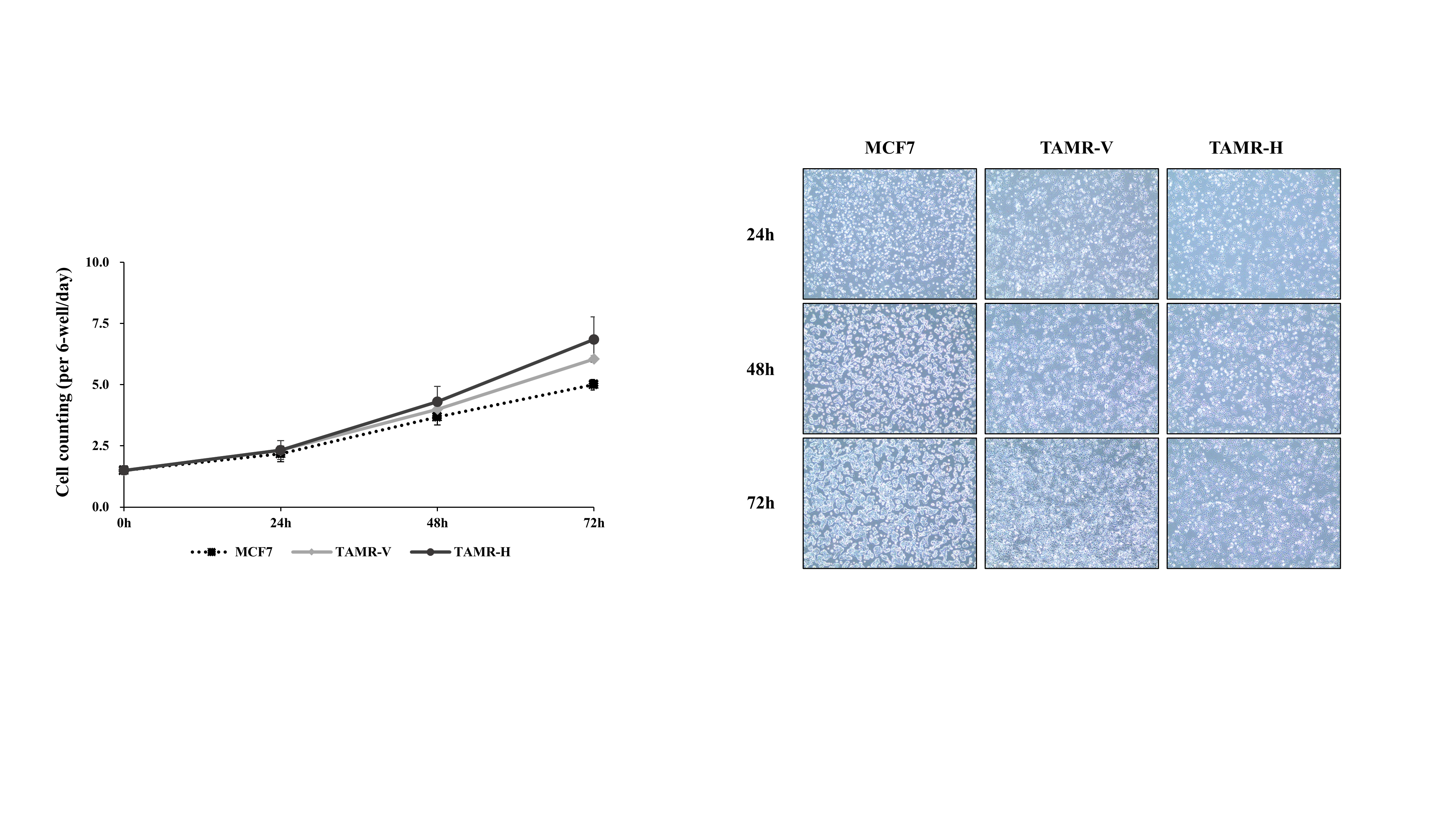

Supplement: Supplementary file 3 — Supplementary Figure 3: Relative expression levels of SOX2OT and SOX2 in various breast cancer cell lines [file 12860_2024_510_MOESM3_ESM.png]
